# *RRP12* Variants Are Associated With Autosomal Recessive Brain Calcifications

**DOI:** 10.1002/mds.70058

**Published:** 2025-10-08

**Authors:** Edoardo Monfrini, Paola Rinchetti, Mathieu Anheim, Anna Klingseisen, Ouhaid Lagha‐Boukbiza, Zhidong Cen, Dehao Yang, Xinhui Chen, Reza Maroofian, Henry Houlden, Gioia Cappelletti, Anne‐Claire Richard, Olivier Quenez, Camilo Toro, Steven J. Frucht, Francesco Lotti, Wei Luo, David Hunt, Gael Nicolas, Giulietta M. Riboldi

**Affiliations:** ^1^ Dino Ferrari Center, Department of Pathophysiology and Transplantation University of Milan Milan Italy; ^2^ Fondazione IRCCS Ca' Granda Ospedale Maggiore Policlinico Neurology Unit Milan Italy; ^3^ Center for Motor Neuron Biology and Diseases, Department of Pathology & Cell Biology and Neurology Columbia University Irving Medical Center New York New York USA; ^4^ Neurology Department Strasbourg University Hospital Strasbourg France; ^5^ Strasbourg Federation of Translational Medicine (FMTS), Strasbourg University Strasbourg France; ^6^ INSERM‐U964; CNRS‐UMR7104 University of Strasbourg Illkirch‐Graffenstaden France; ^7^ UK Dementia Research Institute at University of Edinburgh Edinburgh UK; ^8^ Service de Neurologie Hôpitaux Universitaires de Strasbourg Strasbourg France; ^9^ Department of Neurology The Second Affiliated Hospital, Zhejiang University School of Medicine Hangzhou China; ^10^ Department of Neuromuscular Diseases, Queen Square, Institute of Neurology University College London London UK; ^11^ Department of Biomedical and Clinical Sciences University of Milan Milan Italy; ^12^ Univ Rouen Normandie, Normandie Univ, Inserm U1245 and CHU Rouen Department of Genetics F‐76000 Rouen France; ^13^ National Institutes of Health Undiagnosed Diseases Program, National Human Genome Research Institute National Institutes of Health Bethesda Maryland USA; ^14^ The Marlene and Paolo Fresco Institute for Parkinson's and Movement Disorders, Department of Neurology NYU Langone Health New York New York USA; ^15^ Department of Medical Laboratory Sciences Hunter College, City University of New York New York New York USA; ^16^ Centre for Clinical Brain Sciences at University of Edinburgh Edinburgh UK

**Keywords:** dystonia, brain calcifications, PFBC, primary familial brain calcification, *RRP12*

## Abstract

**Background:**

Primary brain calcifications are observed in several inherited diseases due to different pathogenic mechanisms, including the disruption of the neurovascular unit, mitochondrial dysfunction, and impaired nucleic acid metabolism.

**Objective:**

The aim of the study was to identify a novel genetic cause of brain calcifications in genetically unresolved cases.

**Methods:**

Exome sequencing data from two unrelated Pakistani patients with generalized dystonia and primary brain calcifications were analyzed. The best candidate gene (ie, *RRP12*) was then investigated in two large cohorts of patients with brain calcifications from France (n = 111) and China (n = 543). RRP12 loss‐of‐function phenotype was explored through Western blot and immunocytofluorescence studies on patient‐derived fibroblasts and in a knockdown zebrafish model.

**Results:**

A combined approach of exome sequencing and homozygosity mapping allowed the prioritization of a rare homozygous variant in *RRP12* (c.1558C>T, p.R520C) in two apparently unrelated Pakistani patients from consanguineous families, presenting with infantile‐onset generalized dystonia, spasticity, and widespread brain calcifications. Screening of two large cohorts of patients with unresolved brain calcifications revealed two affected French siblings and one unrelated Chinese individual, each carrying rare, biallelic, missense variants in the *RRP12* gene (c.1429G>A, p.E477K and c.2634T>G, p.F878L, respectively). Molecular studies revealed a significant reduction in RRP12 protein and abnormal nucleolar morphology in patient'derived fibroblasts. Consistent with its essential role in RNA metabolism, rrp12 knockdown in zebrafish caused severe developmental delay, crimping, and early lethality.

**Conclusions:**

*RRP12* is a novel candidate gene for autosomal recessive brain calcifications, possibly associated with a wide clinical spectrum ranging from early‐onset severe forms to adult‐onset paucisymptomatic presentations. © 2025 The Author(s). *Movement Disorders* published by Wiley Periodicals LLC on behalf of International Parkinson and Movement Disorder Society.

Brain calcifications can be primary (idiopathic or genetic) or secondary to other pathological conditions (calcium‐phosphate metabolism disorders, autoimmune disorders, and infections among others). Among the monogenic forms, a growing number of inherited disorders have been associated with intracranial calcifications, arising from a variety of pathogenic mechanisms. These include disruption of the neurovascular unit, leading to impaired blood–brain barrier integrity and dysregulated calcium‐phosphate homeostasis; mitochondrial dysfunction, which alters cellular energy metabolism and intracellular calcium handling; and defects in nucleic acid metabolism, which may trigger neuroinflammatory responses and downstream mineral deposition.

In the context of primary familial brain calcification (PFBC), several causative genes have been identified. *SLC20A2*, *PDGFB*, *PDGFRB*, and *XPR1* are inherited in an autosomal dominant manner, whereas *MYORG*, *JAM2*, and *NAA60* follow an autosomal recessive pattern.^1^, ^2^, ^3^, ^4^, ^5^, ^6^, ^7^, ^8^, ^9^, ^10^ These genes encode for proteins involved in cerebrovascular and blood–brain barrier functions or phosphate transport.^11^ More recently, *CMPK2*, a gene involved in mitochondrial nucleotide synthesis, has been implicated in PFBC or in closely related brain calcification disorder.^12^ Beyond PFBC, numerous other genetic syndromes feature brain calcifications as part of a broader neurological or systemic phenotype. Notably, conditions such as Aicardi‐Goutières syndrome (AGS), leukoencephalopathy with calcifications and cysts (LCC), and Coats plus syndrome are unified by a shared disruption of nucleic acid metabolism. AGS is caused by mutations in genes encoding DNA nucleases, ribonucleases, and cytosolic nucleic acid sensors; LCC is associated with defective ribonucleoprotein biogenesis; and Coats plus results from impaired telomere maintenance.^11^, ^13^, ^14^, ^15^, ^16^, ^17^

In this report, we present *RRP12*, encoding a nucleolar protein involved in RNA metabolism, as a novel candidate gene for autosomal recessive brain calcifications and provide genetic and functional evidence for a causal association.

## Materials and Methods

### Clinical and Genetic Studies

Clinical history and examination, brain imaging studies, and family history were collected by movement disorders or pediatric neurology experts. Genomic DNA was extracted from peripheral venous blood by standard procedures. Probands' DNA was analyzed by exome and/or genome sequencing using commercially available kits on Illumina sequencing platforms, according to the manufacturer's instructions. Reads were aligned against the human reference genome (hg19) using BWA, and variant calling was performed with GATK4.^18^ The candidate variants were validated using Sanger sequencing and tested in available relatives. The tool vcftools‐relatedness2 was used for kinship analysis.^19^ Homozygosity mapping was performed using the AutoMap tool based on exome data.^20^ Copy number variant (CNV) analysis was performed using the Control‐FREEC package.^21^ In silico prediction of the interatomic interactions of the three identified variants was performed with DynaMut.^22^ Written informed consent for genetic analysis and publication of clinical details, clinical images, and videos was obtained from all involved subjects. The studies were approved by Institutional Review Board of each involved center (NYU Movement Disorder Genomic Study, s21‐00207; Rouen University Hospital, Rouen, France, notification E2023‐40; The Second Affiliated Hospital, Zhejiang University School of Medicine, C2020001199; Leeds [East] Research Ethics Committee [10/H1307/132]). The French cohort has already been described in detail elsewhere.^23^

### In Vitro Studies

#### Fibroblast Culture

Fibroblasts from subjects A.II.2, C.II.1, C.II.2, and A.I.2 (healthy mother of A.II.2) were isolated from skin biopsies. An additional line from an unaffected, unrelated, adult male subject was used as a control. All fibroblast lines were cultured in gelatine‐coated (0.1% gelatin, Sigma) plates using Dulbecco's Modified Eagle Medium (DMEM 1X, Corning) supplemented with 15% fetal bovine serum (FBS, Hyclone) and 1% penicillin/streptomycin (Gibco, Thermo Fisher Scientific, 15,070,063). Fibroblast lines were tested for mycoplasma contamination using MycoStrip (Mycoplasma Detection Kit, Invivogen). All the experiments were performed at early passages.

#### Total RNA and Quantitative Real‐Time PCR (q‐RT‐PCR) Analysis

Total RNA was obtained from fibroblasts using the Qiagen RNEasy Kit (Qiagen, 74,106). RNA concentration and quality were checked using a NanoDrop Eight Spectrophotometer (ThermoFisher Scientific). Complementary DNA (cDNA) was generated using SuperScript III reverse transcriptase (Thermo Fisher Scientific, 18,080,044). For the reaction, 1 μg of total RNA was used. The expression level of the candidate genes was assessed using SYBR Green quantitative analysis on the QuantStudio3 Instrument (Applied Biosystems). GAPDH was used as a reference gene. The complete list of the primers is summarized in Table S1.

#### Western Blot Analysis

Cells were lysed in RIPA buffer (G‐Biosciences, 786–489) supplemented with protease and phosphatase inhibitors (Pierce Rockford, IL, USA), passed through a 27‐gauge needle five times, briefly sonicated, and left on ice for 30 minutes. Cell lysates were centrifuged at 16,000*g* for 20 minutes at 4°C, and the supernatant was collected. Protein extracts were quantified using the RC DC protein assay (Bio‐Rad). A total of 30 μg of protein was separated on a 3%–8% NuPAGE Tris‐Acetate gel (Thermo Fisher Scientific) and transferred to nitrocellulose membranes using the iBlot 2 system (Thermo Fisher). Membranes were blocked for 1 hour at room temperature in 5% nonfat dry milk prepared in phosphate‐buffered saline (PBS) containing 0.5% Tween‐20. Primary antibodies (see Table S2) and HRP‐conjugated secondary antibodies (Jackson ImmunoResearch) were diluted in PBS with 0.5% Tween‐20. Proteins were detected using SuperSignal chemiluminescent substrate (Thermo Scientific), according to the manufacturer's instructions. RRP12 antibody (sc‐398,593, Santa Cruz) specificity was validated by small interfering RNA (siRNA)‐mediated knockdown of *RRP12* using a gene‐specific siRNA.

The signal for RRP12 was acquired using the iBright Imaging System (Invitrogen), whereas RRP12 siRNA knockdown, RRP12 mutants, and NOC3L were visualized by autoradiography using Full Speed Blue sensitive X‐ray film (Ewen Parker X‐Ray Corporation). Densitometric analysis was performed using *Image Studio Lite* version 5.2.5.

#### Immunofluorescence

Immunofluorescence analyses were performed according to standard protocols. Briefly, cells were fixed using 4% paraformaldehyde at room temperature for 8 minutes. Blocking was performed for 1 hour using 10% Normal Goat Serum (Jackson ImmunoResearch, AB_2336990) in 1 × PBS/0.25% Tryton. Primary antibodies (Table S2) were diluted in the blocking solution overnight at 4°C. The next day, a secondary antibody (AlexaFluor 488 conjugated, 1:1000; Invitrogen) was added with DAPI (1:500; Sigma‐Aldrich, D9542) in the blocking solution at 1 hour room temperature. Coverslips were mounted on slides using DAKO mounting media (Agilent, S3023). Anti‐RRP12 antibody specificity was validated through knockdown and overexpression experiments (Fig. S2).

Confocal images were acquired using a Leica SP8 system. For each sample, 10 nonoverlapping fields were randomly selected. The number of nucleoli per cell was quantified by counting nucleolin‐positive structures within the nucleus. Total cell number was determined by DAPI staining. Quantification of nucleolar count was performed in a blinded manner after randomization and relabeling of the images with sequential IDs. Cell count was performed counting the DAPI‐labeled nuclei. Nuclei that were partially included in the slide on the border were not included in the final count. Nucleoli were counted using the nucleolin‐labeled slides.

#### Growth Curve

Fibroblasts were cultured in DMEM 1× (Corning, 10‐014‐CV), 15% FBS (Gibco, 26,140,079) and 1% penicillin/streptomycin (Gibco, 15,140,122). At day 0 (D0), cells for each cell line were harvested using Trypsin/EDTA (0.25%, Gibco, 25,200,056) and manually counted. A total of three 10‐cm dishes per line were used, and 1 × 105 cells were plated in each 10‐cm dish. Cells were harvested and counted using ADAM‐MC automated cell counters on day 1 (D1), day 3 (D3), and day 5 (D5).

### Zebrafish Model

Adult male and female wild‐type (WT) and transgenic zebrafish strains were maintained under standard laboratory conditions on a 14‐h light/10‐h dark cycle at 28°C. Experiments were performed in compliance with ethical regulations under Home Office license PL 60/4418. A single orthologue of human *RRP12* is present in zebrafish (*rrp12*). Knockdown of *rrp12* and *noc3l* was performed using target morpholinos designed to target unique genomic regions within the *rrp12* (rrp12 MO) and *noc3l* (noc3l MO) genes. Morpholino sequences were designed by Gene Tools, and clear targeting of the gene of interest was verified through the recommended BLAST search. The following sequences were utilized: standard control MO sequence CCTCTTACCTCAGTTACAATTTATA; noc3l MO sequence GGCCCATTTTGAGCGATGATTTGAC complementary to its translation blocking target (GTCAAATCATCGCTCAAAATGGGCC); rrp12 MO sequence CGGTGGAAATCTTAACTGTGCTGCA, complementary to its translation blocking target (TGCAGCACAGTTAAGATTTCCACCG). A 2 ng/pl concentrated morpholino solution was injected into one‐cell‐stage embryos (3 ng of final injection volume). Direct evidence of rrp12 knockdown in zebrafish could not be obtained, as commercially available antibodies against the human RRP12 protein do not cross‐react with the zebrafish ortholog. Survival was measured each day for the first 6 days for a total of approximately 790 embryos (uninjected control n = 203, control MO n = 163, noc3L MO n = 176, rrp12 MO n = 249). Morphology was documented with bright field imaging collected each day for each group (rrp12 MO, noc3l MO, control MO, uninjected).

### Data Analysis and Statistics

Statistical analyses were performed using GraphPad Prism9. A group comparison was performed using one‐way analysis of variance (ANOVA) followed by multiple comparisons (Dunnett's test). Statistical significance was represented by asterisks according to their calculated probability (*P*‐value). One asterisk indicated a *P*‐value <0.05, and two asterisks indicated a *P*‐value <0.0001.

## Results

### Patient A.II.2

#### Clinical Features

**FIG. 1:**
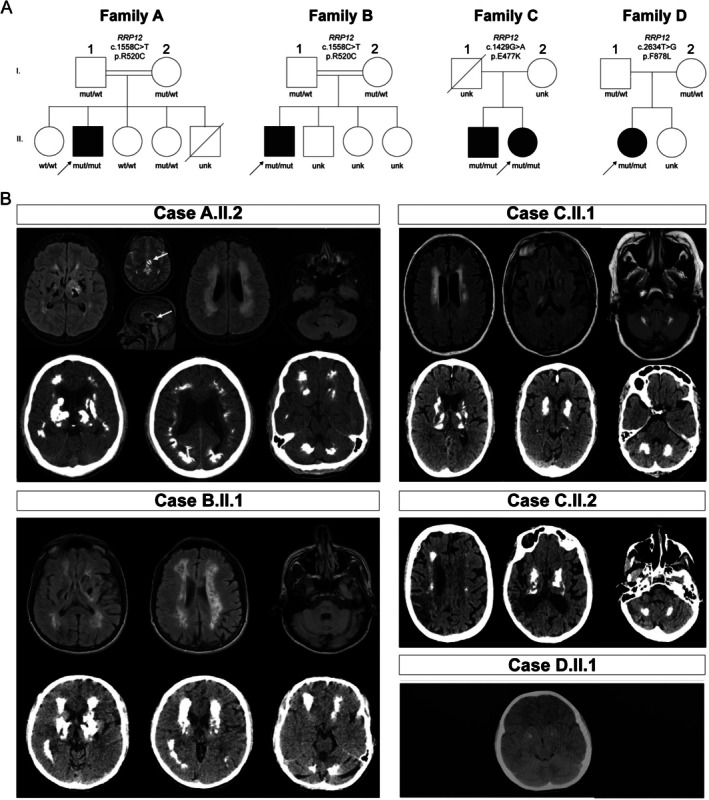
Clinical characterization in *RRP12* homozygous carriers. (**A**) Family trees of the four probands (A.II.2 from Family A, B.II.1 from Family B, both sharing the same p.R520C homozygous variant, and C.II.1 and C.II.2, both being homozygous for the p.E477K variant, D.II.1 from family harboring the homozygous variant p.F778L). Affected individuals are symbols filled in black, whereas the arrows point to probands. Genotypes for the *RRP12* variants are reported below the symbols representing each family member (mut = mutated, wt = wild type, unk = unknown). (**B**) Upper part of panels from cases A.II.2, B.II.1, and C.II.1: brain magnetic resonance imaging (MRI) (T2 FLAIR sequence) showing periventricular white matter hyperintensities along with basal ganglia hypointensities. In patient A.II.2, the arrow indicates a left‐sided thalamic cyst. The lower part of the panel from cases A.II.2, B.II.1, C.II.2 and full panel from cases C.II.2 and D.II.1: brain computed tomography (CT) scan of each affected individual in this study showing extensive calcification in the dentate nuclei, basal ganglia, cerebellum, and supratentorial white matter in cases A.II.2, B.II.1, C.II.1, C.II.2, and faint bilateral lenticular calcifications in case D.II.1.

The first proband was recruited at NYU Langone Health (New York, NY, USA) as part of an undiagnosed disease program. He is a 21‐year‐old Pakistani man who presented with asymmetric generalized dystonia, spasticity, microcephaly, and seizures. He was born at 36 weeks of gestation due to premature rupture of the membranes. He developed seizures at 2 weeks of age, with brain magnetic resonance imaging (MRI) and video electroencephalogram (EEG) reportedly unrevealing. He sat at 9 months and ambulated at 16 months. At the age of 2.5 years, he developed a prolonged focal seizure. A new brain MRI showed abnormal signal in the thalami, lentiform nuclei, and right caudate nucleus consistent with calcifications, decreased frontoparietal subcortical white matter signal in T2 sequence, and a patchy periventricular subcortical white matter signal. EEG was reportedly abnormal. Polymerase chain reaction (PCR) testing for cytomegalovirus (CMV) immunoglobulin G (IgG) was positive, whereas CMV IgM and PCR for herpes simplex virus 1(HSV1) and HSV2 were negative. Congenital CMV infection was initially suspected. At the age of 5 years, impaired coordination, reduced manual dexterity, and learning difficulties prompted transfer to a special aid class. Right eye cataract was diagnosed at the age of 5 years and removed by the age of 6 years. Dystonia of the right limbs was initially noticed at the age of 11 years and gradually progressed to the other side. He started using a wheelchair at the age of 14 and progressively lost the ability to speak, possibly due to laryngeal dystonia and/or pseudobulbar palsy (Video 1 segment 1). He was treated with levodopa, without clinical improvement, baclofen, with partial benefit but he developed headache, and botulinum toxin injections, with benefit on dystonia. Family history was remarkable for consanguinity but not for neurological diseases (Fig. 1A) (Table 1). One of his sisters reported severe combined immune deficiency (SCID), and one brother died from prematurity complications.

**VIDEO 1:** Clinical phenotype of patients A.II.2, B.II.1, and C.II.2. Patient A.II.2 is able to understand and try to perform simple commands; presents with anarthria, but no limitation or abnormalities in eye movements (not shown). There is a severe dystonia of the upper limbs, more pronounced on the right side. Patellar reflexes were increased on both sides. Patient was able to stand and make some steps with support, showing profound dystonia and spasticity of the lower limbs. Patient A.II.1 also presents with anarthria and significant dystonia of both the upper and lower limbs, more severe on the right than the left. There was an increased tone at the knee and a bilateral clonus at the ankle. Patient was able to stand with support but was able to make only a few steps with difficulties. Patient A.C.2 shows preserved speech and facial expression. Choreic movements are observed in the face and distal upper limbs. There is mild bradykinesia with decrement in bilateral upper limbs. When standing with support, she demonstrates a parkinsonian gate with significant gait freezing and small steps.

**TABLE 1:** Demographic and clinical characterization of probands in families A, B, C, and D

	Case A.II.2	Case B.II.1	Case C.II.1	Case C.II.2	Case D.II.1
Genetic variant	c.1558C>T, p.R520C (homozygous)	c.1558C>T, p.R520C (homozygous)	c.1429G>A, p.E477K (homozygous)	c.1429G>A, p.E477K (homozygous)	c.2334 T>G, p.F778L (homozygous)
Ethnicity	Pakistani	Pakistani	European (France)	European (France)	Chinese
Consanguinity	Yes	Yes	UKN	UKN	No
Gender	Male	Male	Male	Female	Female
Age at onset	2 w	6 y	63 y	59 y	10 y
Age at evaluation	16 y	28 y	63 y	66 y	14 y
Symptom at onset	Seizures	Dysarthria, stuttering	None	Bipolar disorder	Dizziness
Dystonia (onset)	Yes (11 y)	Yes (6 y)	/	/	/
Spasticity (onset)	Yes (NA)	Yes (6 y)	/	/	/
Parkinsonism (onset)	/	/	/	Yes (60 y)	/
Ataxia (onset)	/	/	/	Yes (60 y)	/
Chorea (onset)	/	/	/	Yes (60 y)	/
Cognitive impairment (onset)	NA	NA	/	Yes (60 y)	/
Psychiatric symptoms (onset)	/	/	Anxiety (20)	Bipolar (59 y)	/
Tinnitus (onset)	/	/	Yes (50)	/	/
Other	Microcephaly, cataract	Thrombocytopenia	/	/	/
Brain imaging	Calcifications (thalami, lentiform nuclei, right caudate), periventricular leukoencephalopathy, one cyst	Calcifications (cerebellum, midbrain, deep white matter), diffuse leukoencephalopathy, mild brain atrophy	Calcifications (cerebellum, striatum, thalamus), leukoencephalopathy	Calcifications (cerebellum, striatum, thalamus, hippocampus), leukoencephalopathy	Calcifications (lentiform nuclei)
TCS	57	60	41	46	4

*Note*: Genetic and clinical features of the affected subject in the four families are reported.

Abbreviations: UKN, unknown; NA, not available; TCS, total calcium score.

Metabolic tests (parathyroid hormone, calcium, magnesium, manganese, iron, total iron binding capacity, renal and hepatic panel, thyroid‐stimulating hormone [TSH], lactate, pyruvate, complete blood count, plasma amino acids, urine organic acids, pterins on blood and urine, comprehensive acylcarnitine panel, very‐long‐chain fatty acids), liver ultrasound, X‐rays of hands and feet were unrevealing. Brain MRI at the age of 15 years showed extensive areas of calcification (cerebral hemispheres, bilateral basal ganglia, thalami, and bilateral dentate nuclei), extensive confluent white matter changes in both cerebral hemispheres, and one intracerebral cyst in the ventral medial left thalamus (Fig. 1B). A brain computed tomography (CT) scan at the age of 18 years showed marked, bilateral, symmetric calcifications of the subcortical white matter, basal ganglia, and dentate nuclei (total calcification score [TCS] = 57). At that point, a congenital CMV infection was deemed insufficient to explain the patient's progressive clinical phenotype, so he underwent extensive genetic tests.

#### Genetic Analyses

**FIG. 2:**
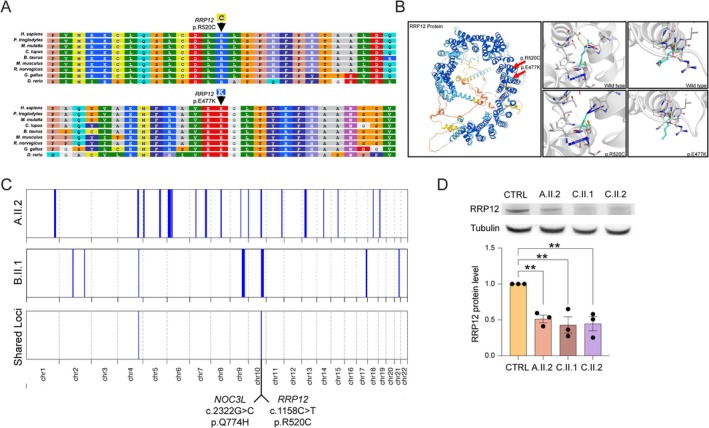
Genetic and molecular characterization of *RRP12* gene variants. (**A**) *RRP12* p.(R520C) and p.(E477K) variants are highly conserved across species. (**B**) In silico analyses (DynaMut and ColabFold) predict the destabilization of mutated RRP12 proteins. Wild‐type and mutant residues are colored in light green and are also represented as sticks alongside the surrounding residues, which are involved in any type of molecular interaction. (**C**) Homozygosity mapping of probands A.II.2 and B.II.1 showing shared loci of homozygosity on chromosome 4 (hg19: Chr4:155,525,695‐159,727,192) and chromosome 10 (Chr10:95,360,964‐101,150,256), which includes the *RRP12* c.1558C>T and *NOC3L* c.2322G>C variants. (**D**) Western blot analysis of RRP12. Quantification of RRP12 revealed a statistically significant decrease in RRP12 in all affected samples compared to the control (*P* = 0.0029). *P ≤ 0.05; **P ≤ 0.01; ***P ≤ 0.001.

**FIG. 3:**
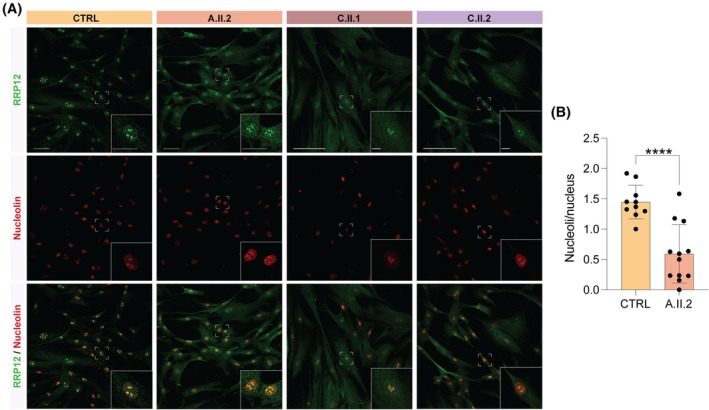
Colocalization of RRP12 and nucleolar markers. (**A**) Representative immunofluorescence of RRP12 (green) and nucleolin (red) in the control and affected samples. Nucleolar structures are less defined and morphologically disrupted in patients' fibroblasts (A.II.2, C.II.1, C.II.2) compared to the control. Scale bar: 50 and 25 μm. (**B**) Quantification of number of nucleoli per cell in proband A.II.2 compared to control (*P* = <0.0001). *P ≤ 0.05; **P ≤ 0.01; ***P ≤ 0.001.

Peripheral blood karyotype was normal. High‐density array‐CGH did not reveal any pathogenic CNVs but identified long runs of homozygosity, consistent with a family history of consanguinity. No candidate genes were located within these regions. Trio exome (A.II.2, A.I.I, and A.I.2), including mitochondrial genome analysis, from 2018 was unrevealing. Because the clinical phenotype of the patient resembled LCC, Sanger sequencing of *SNORD118* (not covered by exome) was performed, including 5′ and 3′ regions in the proximity of the gene. No rare candidate variants were detected. Trio exome data were subsequently reanalyzed, and filtering, based on allele frequency and predicted protein impact, yielded 622 nonsynonymous variants, none affecting genes already associated with brain calcifications, microcephaly, epilepsy, or cataracts. Considering the high degree of consanguinity in the family, an additional filter for homozygous variants was applied, resulting in 33 variants. Among these homozygous variants, only 5 were heterozygous/absent in the healthy parents and siblings (Table S3). The *RRP12* variant [NM_015179.4: c.1558C>T, p.(R520C)] was prioritized as the best candidate for the following reasons: (1) it is predicted as the most deleterious in silico (eg, MetaRNN 0.9758, CADD 32, DANN 0.9989, SIFT‐PROVEAN −7.72); (2) it affects a highly conserved amino acid residue among orthologues (Fig. 2A); (3) it is extremely rare in population databases (gnomAD v4.1.0 AF = 0.00002852, no homozygous carrier, GenomeAsia AF = 0.000287, no homozygous carrier, UK Biobank AF = 1.36E‐06, no homozygous carrier; Genomics England Research Environment—GEL—aggregated variant calls (AggV2) AF = 9/156390, no homozygous carrier); (4) *RRP12* is expressed widely in human tissues, including blood vessels and brain; (5) *RRP12* displays mild intolerance to missense variants (gnomAD o/e = 0.89 [0.85–0.93]) and moderate intolerance to loss‐of‐function (LoF) variants (gnomAD o/e = 0.46 [0.37–0.56]); (6) RRP12 like (RRP12), the protein encoded by *RRP12*, is involved in nucleic acid metabolism, as several other genes associated with other genetic forms of brain calcifications. This variant was localized in a homozygous stretch on chromosome 10, in line with the known consanguinity of the parents, who are both heterozygous and unaffected (Fig. 2B). Finally, trio whole‐genome sequencing (WGS) was performed to rule out cryptic variants in genes implicated in brain calcifications, leukoencephalopathy, and LCC. No pathogenic variants were detected. CNV analysis identified one significant homozygous loss (chr4:156,959,215‐156,974,013) in the proband on chromosome 4, which was also shared in homozygous status by the parents.

### Patient B.II.1

#### Clinical Features

After the *RRP12* variant was identified in the first family, we contacted the colleagues at the Wessex Clinical Genetics Service, Princess Anne Hospital (Southampton, UK), to inquire about additional cases with biallelic *RRP12* variants, given their expertise in LCC‐like phenotypes. Notably, they were investigating a young Pakistani male (B.II.1) who presented with a phenotype strikingly similar to case A.II.2, in whom the same homozygous *RRP12* variant, c.1558C>T, p.(R520C), had been prioritized. Patient B.II.1 is a 28‐year‐old man with infantile‐onset progressive generalized dystonia, spasticity, and severe speech impairment accompanied by extensive brain calcifications and white matter changes. According to the parents and clinical reports, the patient experienced mild perinatal asphyxia without evident clinical consequences, and early psychomotor development was normal until age 6, when dysarthria and stuttering began to appear. Throughout his teenage years, he gradually developed a progressive dystonic‐spastic asymmetric quadriparesis, worse on the right, pseudobulbar palsy with emotional lability, and anarthria. AGS was suspected, but assessment of the expression of interferon‐stimulated genes (ISGs) in blood was unremarkable, and evaluation of known AGS genes using Sanger sequencing was negative. At the age of 20 years, he became wheelchair bound, needing assistance with feeding and toileting. Brain CT and MRI performed at the age of 24 years showed extensive parenchymal calcifications affecting cerebral deep white matter, cerebellum, midbrain, diffuse white matter changes, and mild global brain atrophy (Fig. 1B, Video 1 segment 2). Spine MRI, abdomen ultrasound imaging, and blood tests were unrevealing except for mild thrombocytopenia. Parents were consanguineous (first cousins) (Fig. 1A) (Table 1). He had three healthy siblings and no history of neurological disorders.

Considering the overlapping phenotypes of A.II.2 and B.II.1, the history of consanguinity in both patients, and the common Pakistani ancestry, the exome raw data of both subjects were reanalyzed jointly under the hypotheses of distant relatedness and of a shared homozygous mutation causing the common phenotype. In line with our hypothesis, bioinformatic analyses revealed a third degree of kinship between the two probands. Homozygosity mapping showed two haploidentical homozygous regions shared by patients on chromosomes 4 (Chr4:155,525,695–159,727,192 bp) and 10 (Chr10: 95,360,964–101,150,256) (Fig. 2C). Only two rare homozygous variants shared by both patients were localized in these regions: *RRP12* c.1558C>T, p.(R520C) and *NOC3L* c.2322G>C, p.(Q774H) (Fig. 2C).

*NOC3L* encodes for the NOC3‐like DNA replication regulator protein, reported to be involved in adipogenesis, RNA binding, and DNA replication. The *NOC3L* missense variant is very rare (gnomAD v4.1.0 AF = 0.00002235), is predicted benign by the majority of in silico tools (eg, MetaRNN 0.03123, CADD 0.032, DANN 0.8175, SIFT‐PROVEAN‐1.44), and affects a nonconserved amino acid. For these reasons, *NOC3L* was considered a much less compelling candidate gene compared to *RRP12*.

### *RRP12* Screening in Cohorts of Patients with Brain Calcifications

To corroborate the role of *RRP12* as a possible novel disease gene, we interrogated two datasets of exome data of patients with unresolved brain calcifications in France (Rouen) and China (Zhejiang). Overall, three additional subjects from two families exhibited rare biallelic coding *RRP12* variants.

#### French Cohort: Patients C.II.2 and C.II.1

Among 111 probands with unresolved PFBC following exome analysis, a rare homozygous *RRP12* variant (c.1429G>A, *P*.E477K) was found in a French woman (C.II.2), with no reported family history of neurological disorders or consanguinity. The identified variant is extremely rare in population databases (gnomAD v4.1.0 AF = 6.197e‐7, no homozygous carrier). Interestingly, the localization of the E477 in the ternary structure of the protein is in close proximity to the R520 position, hinting at a possible functional interaction. Although this variant is predicted to be benign by most in silico prediction tools, it affects a moderately conserved amino acid in orthologs and displays a moderately high CADD score (22.6). No rare biallelic *NOC3L* coding variants were found in the French cohort.

Patient C.II.2 was 59 years old when diagnosed with bipolar disease and, shortly thereafter, developed mild cerebellar ataxia, choreic movements, and cognitive impairment with dysexecutive syndrome and verbal episodic memory abnormalities. She also presented subtle parkinsonism (possibly iatrogenic) with mild bradykinesia and rigidity and normal 123I‐Ioflupane SPECT (Video 1, segment 3) (Table 1). CT scan showed marked, bilateral, symmetric calcifications of the cerebellum, striatum, thalamus, hemispheric white matter, and subtle subcortical calcifications (TCS = 46 at the age of 65 years). Remarkably, her brother (C.II.1), who suffered from mild anxiety and chronic tinnitus, showed a similar calcification pattern as the sister at head CT (TCS = 41 at the age of 61 years) and carried the same homozygous *RRP12* variant, as revealed using Sanger sequencing.

#### Chinese Cohort: Patient D.II.1

Among 543 patients with brain calcifications, one Han Chinese female patient (D.II.1) carried the rare homozygous *RRP12* c.2634 T>G, p.(F878L) variant (Fig. 1) (Table 1). The identified variant is extremely rare in population databases (gnomAD v4.1.0 AF ≤0.000002483, no homozygous carrier). The c.2634 T>G is predicted to be benign by the majority of in silico tools (MetaRNN 0.3097, CADD 0.01499, DANN 0.9642, SIFT‐PROVEAN‐3.59) and affects a moderately conserved amino acid (Fig. S1). No rare biallelic *NOC3L* variants were found in the Chinese cohort. We also searched for this variant in two independent Chinese population datasets—the NyuWa Chinese Population Variant Database (n = 2999) and the Westlake BioBank for Chinese (n = 10,376)—and found no carriers, indicating that the F878L variant is extremely rare in this population. D.II.1 came to medical attention at the age of 10 years, complaining of dizziness. Brain CT showed faint bilateral lenticular calcifications (TCS = 4 at the age of 14) (Fig. 1B).

### *RRP12* Variants Reduce Protein Levels and Are Associated with a Diminished Proliferative Rate and Impaired Nucleolar Structures

Skin‐derived fibroblasts from probands A.II.2, C.II.1, C.II.2, unaffected mother A.I.2 (heterozygous carrier), and healthy control presented normal morphology (Fig. S3A,B). A statistically significant reduction in RRP12 protein levels in the probands' fibroblasts compared to the unaffected controls was detected (Fig. 2D; one‐way ANOVA, WT vs. A.II.2 *P* = 0.0067, WT vs. C.II.1 *P* = 0.0026, WT vs. C.II.2 *P* = 0.0032), whereas messenger RNA (mRNA) levels did not show any statistically significant reduction (Fig. S3C; one‐way ANOVA, WT vs. A.II.2 *P* = 0.0005).

Immunofluorescence studies showed that RRP12 protein was localized in the nucleolus of fibroblasts, as expected.^24^ In patients' fibroblasts, abnormal nucleoli morphology, which appeared to coalesce in disrupted nuclear structures, was observed (Fig. 3A). The number of nucleoli per cell was quantified in control and patient‐derived fibroblasts. A significant reduction in nucleolar number was observed in patient cells compared to controls (Welch's *t* test, *P* < 0.0001), indicating nucleolar abnormalities potentially associated with the underlying genetic condition (Fig. 3B).

Furthermore, A.II.2‐derived fibroblasts showed a diminished proliferative rate compared to the unaffected mother and control cells (Fig. 2D). Fibroblast growth rate is inversely proportional to age,^25^ suggesting an even greater growth defect in the proband if compared to age‐matched controls.

Functional analyses of the *NOC3L* transcript and protein did not reveal any specific abnormalities in mRNA levels and only minimal protein reduction in mutants compared to controls (Fig. S4A,B).

### rrp12 Knockdown Affects the Survival and the Phenotype of Zebrafish

**FIG. 4:**
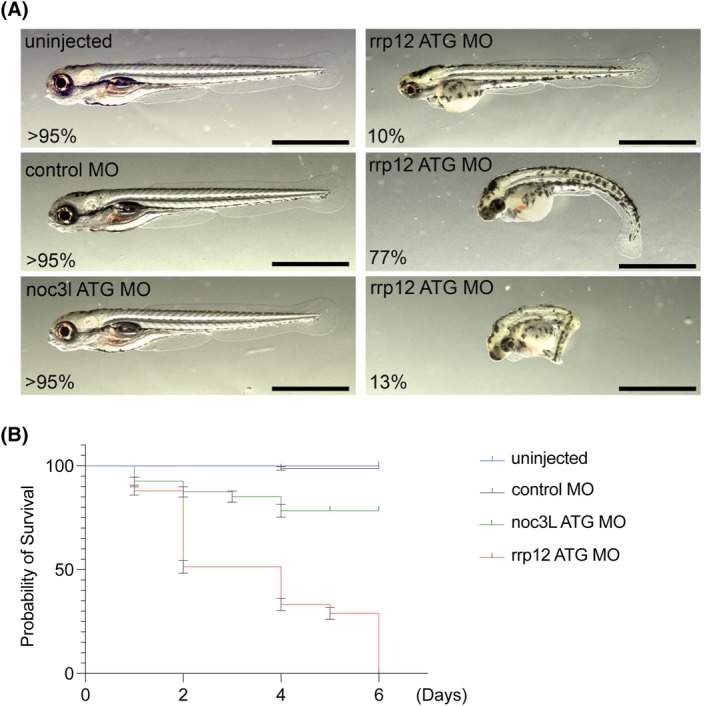
Zebrafish treated with morpholino against rrp12 and noc3l. (**A**) Stage of development at day 7 after morpholino injection in the zebrafish embryos. Top and middle left: uninjected animals and embryos injected with control MO show normal development, normal head size, and straight body. Lower left: embryos injected with noc3l ATG MO also present normal development, size, and body strength. Right: embryos injected with rrp12 ATP MO presented different degrees of abnormalities on day 7, which were consistent with delayed development and significant reduction in head size (10% of embryos, top right), crimping, and developmental delay (77% of embryos, middle right), as well as severe crimping and developmental abnormalities (13% of embryos, lower right). (**B**) Survival was significantly reduced by day 6 in the rrp12‐injected embryos, whereas it was only mildly reduced (to 80%) in noc3l ATG MO‐injected fish. Control MO did not alter survival compared to uninjected animals.

Zebrafish embryos injected with rrp12 MO showed a dramatic reduction in survival compared to embryos injected with control MO and untreated animals. rrp12 MO‐treated embryos showed a 50% survival at 2 days and a maximum survival of 6 days (Fig. 4A). Animals injected with control MO and uninjected zebrafish presented 100% survival at 6 days. For embryos injected with noc3l MO, survival at 6 days was slightly reduced (80%) (Fig. 4B).

Consistently with the survival study, phenotype analysis also showed noticeable abnormalities in the majority of the zebrafish injected with rrp12 MO at 6 days (severe in 77% of animals and very severe in 13% of the animals) compared to animals injected with noc3l MO, ctrl MO, and uninfected animals where more than 95% of the embryos showed normal morphology at day 6. Phenotype abnormalities in the embryos injected with the rrp12 MO consisted of delayed development and crimping of the animals with a range of degrees in severity (Fig. 4A).

## Discussion

We propose the *RRP12* gene as a novel genetic cause of autosomal recessive brain calcification. We identified the candidate homozygous *RRP12* p.(R520C) variant in two unrelated Pakistani patients (A.II.2 and B.II.1). Homozygosity mapping in the two probands supported the pathogenic role of this variant. Two additional homozygous variants [p.(E477K) and p.(F778L)] were found in two French siblings and in one Chinese patient, respectively.

*RRP12* encodes the RRP12‐like protein, which is involved in ribosomal maturation and export from the nucleus and RNA metabolism in the cell.^24^ Interestingly, several genetic disorders characterized by brain calcifications and leukoencephalopathy—including AGS, LCC, and Coats plus syndrome—have been linked to defects in nucleic acid metabolism.^17^ For instance, AGS involves aberrations in DNA nucleases, ribonucleases, and innate immune sensing of nucleic acids; LCC is associated with defects in ribonucleoprotein biogenesis; and Coats plus syndrome involves impaired telomere maintenance.^17^ It is biologically plausible that a perturbation of a similar pathway from different genetic changes may produce similar phenotypes.

Reduced RRP12 protein levels in the patients' fibroblasts may suggest a possible LoF mechanism. The LoF hypothesis is also supported by the proliferative defect of mutated fibroblasts since it has been demonstrated that RRP12 loss is associated with a delay in the M/G1 cell cycle transition^26^ and, on the opposite, that RRP12 overexpression could rescue the cells growth suppression due to doxorubicin treatment.^27^

Importantly, the expression of *RRP12* in vascular cells raises the possibility that it contributes to cerebrovascular homeostasis. In PFBC, several causative genes (eg, *PDGFB*, *PDGFRB*, *SLC20A2*, *XPR1*, *MYORG*) are expressed in the neurovascular unit and are involved in phosphate transport, vascular integrity, and pericyte function.^11^ Thus, one can speculate that *RRP12* dysfunction may converge on shared pathogenic pathways involving nucleic acid metabolism and vascular dysregulation, potentially linking impaired ribonucleoprotein processing to vascular calcification. Further investigation is warranted to determine whether RRP12‐related disease mechanisms intersect with these known pathways in PFBC and related conditions.

Although we could not reproduce the characteristic brain features associated with this condition, the lethal effect of knocking down *rrp12* in a zebrafish model further supports a possible pathogenic role of LoF variants of this gene. In addition, the embryos injected with rrp12 MO presented a delayed development and a crimping appearance. The reported functional studies support a detrimental role of LoF variants in the *RRP12* gene. However, it is important to consider that rrp12 localization, expression pattern, or functional roles in zebrafish may differ from those of the human RRP12 protein. Such species‐specific differences could account for the absence of a brain calcification phenotype in the model, despite evidence of a critical role in development. Therefore, although the current functional studies support the pathogenic potential of RRP12 disruption, further investigation in mammalian systems or patient‐derived cellular models will be essential to clarify its role in disease mechanisms, particularly in the context of brain calcifications.

It is also important to note that brain calcifications are notoriously difficult to detect in animal models and often take several weeks or even months to develop. For instance, in *SLC20A2* mutant mice, calcifications appear only after 15 weeks^28^; in mice with hypomorphic *PDGFB* alleles, they are seen after 2 months^2^; in *PDGFRB* models after 14 months^29^; and in *JAM2* knockout mice not until 18 months of age.^9^ No brain calcifications were found in 19‐month‐old Naa60 knockout mice.^10^ In zebrafish, brain calcifications have been observed as early as 2–4 days postfertilization, but only in *MYORG* models.^30^ However, in MYORG‐knockout mice, calcifications are not reported before 9 months of age.^30^ These findings are consistent with clinical observations, where brain calcifications may not be visible during early development or in the first months of life—as was the case for our first proband (A.II.2).

The phenotypic spectrum of *RRP12* pathogenic variants identified in this work includes an infantile form, classical PFBC phenotype, and paucisymptomatic subjects with prominent brain calcifications. Notably, the clinical features of the two Pakistani probands—including developmental delay, white matter changes, and intracranial calcifications—overlap with features seen in AGS and LCC. Both AGS and LCC are characterized by early‐onset brain calcifications and leukodystrophy. This phenotypic overlap supports the hypothesis that RRP12‐related disorders may extend beyond the classical PFBC spectrum, bridging clinical and radiological features of AGS, LCC, and PFBC. Although a genotype–phenotype correlation may explain the very different age of onset and clinical presentation (ie, possible milder *RRP12* mutations in paucisymptomatic subjects), the limited number of cases imposes further studies. In addition, a modulatory effect of other variants (eg, *NOC3L* variant) or of other comorbidities (eg, CMV infection in patient A.II.2) cannot be ruled out. Consistently, the milder radiological and clinical phenotype observed in individual D.II.1 may reflect the less‐severe nature of the variant identified in this case, although its pathogenic role remains highly uncertain. Further functional studies will be necessary to clarify its contribution to the disease. Moreover, it should be noted that rare biallelic predicted deleterious variants in this gene are uncommon, though not exceedingly rare. Therefore, the finding of biallelic variants in *RRP12* should be interpreted with caution, as the association with phenotype may be spurious.

Considering that the probands from families A and B were of Pakistani origin, and the identified *RRP12* variant (c.1558C>T) is almost completely confined to South Asians (gnomAD v4.1.0), this variant is likely a founder mutation, and other homozygous carriers can be found in this population.

In conclusion, *RRP12* is a novel candidate gene for autosomal recessive brain calcifications, possibly associated with a broad clinical spectrum ranging from early‐onset severe forms to adult‐onset paucisymptomatic presentations.

## Author Roles

(1) Research project: A. Conception, B. Organization, C. Execution; (2) Analysis: A. Design, B. Execution, C. Review and Critique; (3) Manuscript Preparation: A. Writing of the First Draft, B. Review and Critique.

E.M.: 1A, 1B, 1C, 2A, 2B, 2C, 3A, 3B.

P.R.: 1B, 1C, 2A, 2B, 2C, 3A, 3B.

M.A.: 1A, 1B, 1C, 2A, 2C, 3A, 3B.

A.K.: 1C, 2A, 2B, 2C, 3A, 3B.

O.L.B.: 1C, 2B, 2C, 3B.

Z.C.: 1C, 2B, 2C, 3B.

D.Y.: 1C, 2B, 2C, 3B.

X.C.: 1C, 2B, 2C, 3B.

R.M.: 1C, 2B, 2C, 3B.

H.H.: 1B, 2C, 3B.

G.C.: 1C, 2B, 2C, 3B.

A.C.R.: 1C, 2B, 2C, 3B.

O.Q.: 1C, 2B, 2C, 3B.

C.T.: 1A, 1B, 2C, 3B.

S.J.F.: 1A, 1B, 2C, 3B.

F.L.: 1A, 1B, 2A, 2C, 3B.

W.L.: 1A, 1B, 2C, 3B.

D.H.: 1A, 1B, 2C, 3A, 3B.

G.N.: 1A, 1B, 2C, 3A, 3B.

G.M.R.: 1A, 1B, 1C, 2A, 2C, 3A, 3B.

## Financial Disclosures of all authors (for the Preceding 12 Months)

Nothing to disclose.

## Supporting information


**Data S1.** Supporting information.

## Data Availability

The data that support the findings of this study are available from the corresponding author upon reasonable request. Individual‐level exome sequencing data are not available for sharing as they represent personal data.
